# Emerging Roles of GDF15 in Metabolic and Cardiovascular Diseases

**DOI:** 10.34133/research.0832

**Published:** 2025-08-19

**Authors:** Tian Tian, Monan Liu, Peter J. Little, Hans Strijdom, Jianping Weng, Suowen Xu

**Affiliations:** ^1^ Department of Endocrinology, Centre for Leading Medicine and Advanced Technologies of IHM, The First Affiliated Hospital of USTC, Division of Life Sciences and Medicine, University of Science and Technology of China, Hefei 230001, China.; ^2^ Anhui Provincial Key Laboratory of Metabolic Health and Panvascular Diseases, Hefei 230001, China.; ^3^ School of Pharmacy and Pharmaceutical Sciences, The University of Queensland, Woolloongabba, QLD4102, Australia.; ^4^ Centre for Cardio-metabolic Research in Africa, Division of Medical Physiology, Faculty of Medicine and Health Sciences, Stellenbosch University, Cape Town, South Africa.

## Abstract

Growth differentiation factor 15 (GDF15), a TGF-β superfamily member and stress-responsive cytokine, plays a critical role in metabolism and regulation of inflammation. This review summarizes the expression, distribution, structure, processing, and secretion of GDF15. We also discuss multilayered regulatory networks governing GDF15 expression, including ATF4/CHOP, AMPK, EGR1, EZH2, PPARγ, NRF2, ERRγ, and p53, as well as posttranscriptional regulator CNOT6L. The GFRAL receptor is central to its function, mediating the anorexigenic and metabolic effects of GDF15. This review synthesizes evidence linking GDF15 to obesity, diabetes, cardiovascular diseases, metabolic liver disorders, cachexia, sarcopenia, and aging while exploring its interactions with key metabolic factors including FGF21, GLP-1, leptin, and glucocorticoids. Lifestyle interventions such as ketogenic, high-fat diets, and exercise modulate GDF15 levels, underscoring its role in pan-metabolic health. Pharmacologically, various agents—including anti-hyperglycemic agents and natural compounds—induce GDF15 expression, implicating their therapeutic potential in cardiometabolic diseases. We comprehensively evaluate current advances in GDF15-targeted drug development, including monoclonal antibodies, fusion proteins, and small-molecule drugs, to provide a scientific foundation for innovative therapies. Finally, we outline unresolved issues in GDF15 biology and therapeutics, such as the exploration of peripheral receptors, contradictory findings in studies of cardiometabolic diseases, and the persistent challenges in developing GDF15-targeted therapeutics.

## Introduction

Growth differentiation factor 15 (GDF15) is a member of transforming growth factor-β (TGF-β) superfamily of pleiotropic proteins [1]. This secreted protein forms disulfide-linked homodimers and serves as a crucial regulator of diverse biological processes, including cellular stress response, energy homeostasis, and inflammation [2]. Its expression is induced in pathological states, positioning it as a crucial mediator in disease pathogenesis [2].

As a secreted protein, GDF15 is implicated in a variety of prevalent and incident diseases and is frequently regarded as a risk factor for these conditions [3]. Given its extensive associations with multiple pathological states, GDF15 is recognized as a biomarker of notable clinical utility [3]. Cardiometabolic disorders, encompassing both cardiovascular and metabolic diseases, represent a serious public health and economic burden owing to their widespread prevalence and lethal outcomes [4]. These disorders often share common etiological pathways, such as chronic inflammation, insulin resistance, and endothelial dysfunction. GDF15 has gained attention for its potential to bridge these interconnected pathways. Increased circulating GDF15 levels have been consistently associated with adverse outcomes in obesity-related metabolic disorders, heart failure, and atherosclerosis, indicating its diagnostic and prognostic significance and potential [5]. The diverse roles of GDF15 in both cardiovascular and metabolic diseases underscore its therapeutic potential. Currently, several drugs targeting the GDF15/GFRAL axis are in clinical development for various indications, including obesity, heart failure, and cachexia. These advances hold promises for novel treatment strategies and better prognosis managements.

In this review, we aim to provide a comprehensive overview of the emerging understanding of GDF15 in cardiovascular and metabolic diseases, highlighting its mechanistic and biological regulation, clinical implications, and therapeutic potential. This review seeks to highlight the translational potential of GDF15 and identify gaps in knowledge that warrant further investigation.

## Expression and Distribution of GDF15

The distribution and expression levels of GDF15 differ across species and tissues. In mice, GDF15 is predominantly present in the liver, kidneys, and adipose tissue under physiological conditions [6–8]. Metabolic stress induced by obesity triggers GDF15 secretion from hepatic and adipose sources, leading to increased circulating levels [9]. In humans, however, the placenta demonstrates the highest expression levels among all tissues examined, while the lung, kidney, colon, and liver exhibit lower levels of expression [10]. Quantitative analysis somewhat surprisingly reveals no marked difference of GDF15 mRNA levels in adipose tissue between lean and obese individuals [11]. This suggests that up-regulation in circulating GDF15 observed in obesity may not arise from adipose tissues in humans. However, a recent study revealed that lipid-associated macrophages (LAMs) in brown adipose tissue (BAT) secrete GDF15 to maintain BAT homeostasis [12]. Immune cells such as granulocytes and monocytes, which express GDF15 and are activated during inflammation, may contribute, particularly in the liver where immune cell infiltration occurs in obesity [13]. Single-cell transcriptional profiling of human liver biopsies revealed that GDF15 expression increases across various liver cell types, especially cholangiocytes and endothelial cells, in conditions like cirrhosis and metabolic dysfunction-associated steatohepatitis (MASH) [14,15]. During pathological events such as myocardial infarction, ischemia–reperfusion injury, and metabolic disorders, GDF15 expression increases in multiple tissues, including the kidney [8,16], heart [17], skeletal muscles [18], and adipose tissue [19]. The broad tissue distribution highlights the essential role of GDF15 in adapting and responding to diverse stress conditions. Further research is needed to explore their relevance and identify the key cells and tissues contributing to elevated circulating GDF15.

## Structure, Processing, and Secretion of GDF15

**Fig. 1.**
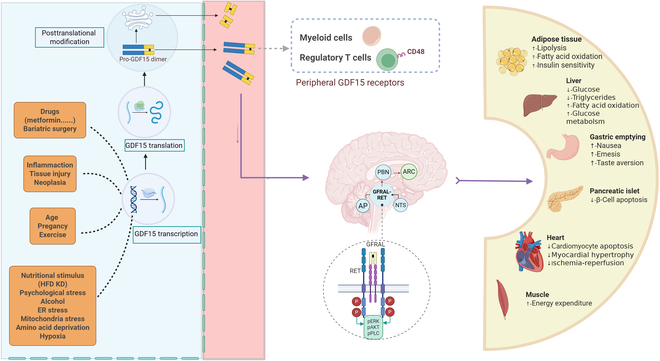
The synthesis, secretion, and biological functions of GDF15. Under various stimuli, GDF15 transcription is induced, followed by translation and posttranslation modification of GDF15. GDF15 exerts multifaceted cadiometabolic regulation in mice through GFRAL binding in the area postrema (AP) and nucleus of the solitary tract (NTS) regions. Furthermore, myeloid cells and regulatory T cells could express peripheral receptors of GDF15. ER, endoplasmic reticulum; HFD, high-fat diet; KD, ketogenic diet; ARC, arcuate nucleus; PBN, parabrachial nucleus; pERK, phosphorylated extracellular signal-regulated kinase; pAKT, phosphorylated protein kinase B; pPLC, phosphorylated phospholipase C.

As a member of the TGF-β superfamily, GDF15 exhibits pleiotropic biological activities. GDF15 shares limited homology with both the glial cell line-derived neurotrophic factor (GDNF) family and classical TGF-β members [20]. The GDF15 gene, which is located on chromosome 19p13.11, displays a characteristic 2-exon architecture separated by one intron [21]. Molecular analysis reveals that its product is initially translated as a 308-residue precursor protein (predicted molecular weight: 35 kDa) containing an N-terminal 29-amino acid signal sequence, followed by the prodomain and mature domain regions [22]. The Furin enzyme subsequently cleaves the precursor peptide from the mature peptide at the RXXR site. The latter, which is the biologically active form of GDF15, exists in a dimeric state upon secretion containing 224 amino acids and has a molecular weight of approximately 25 kDa [20,22]. Once mature, GDF15 is secreted from the cell via the classical secretory pathway. It is packaged into vesicles within the Golgi apparatus and then released into the extracellular space through exocytosis [23,24]. GDF15 can be found in both soluble and membrane-bound forms, with the soluble form being the primary mediator of its biological effects [24]. The synthesis and secretion process of GDF15 are depicted in Fig. 1.

## Multifaceted Regulation of GDF15 Expression

**Fig. 2.**
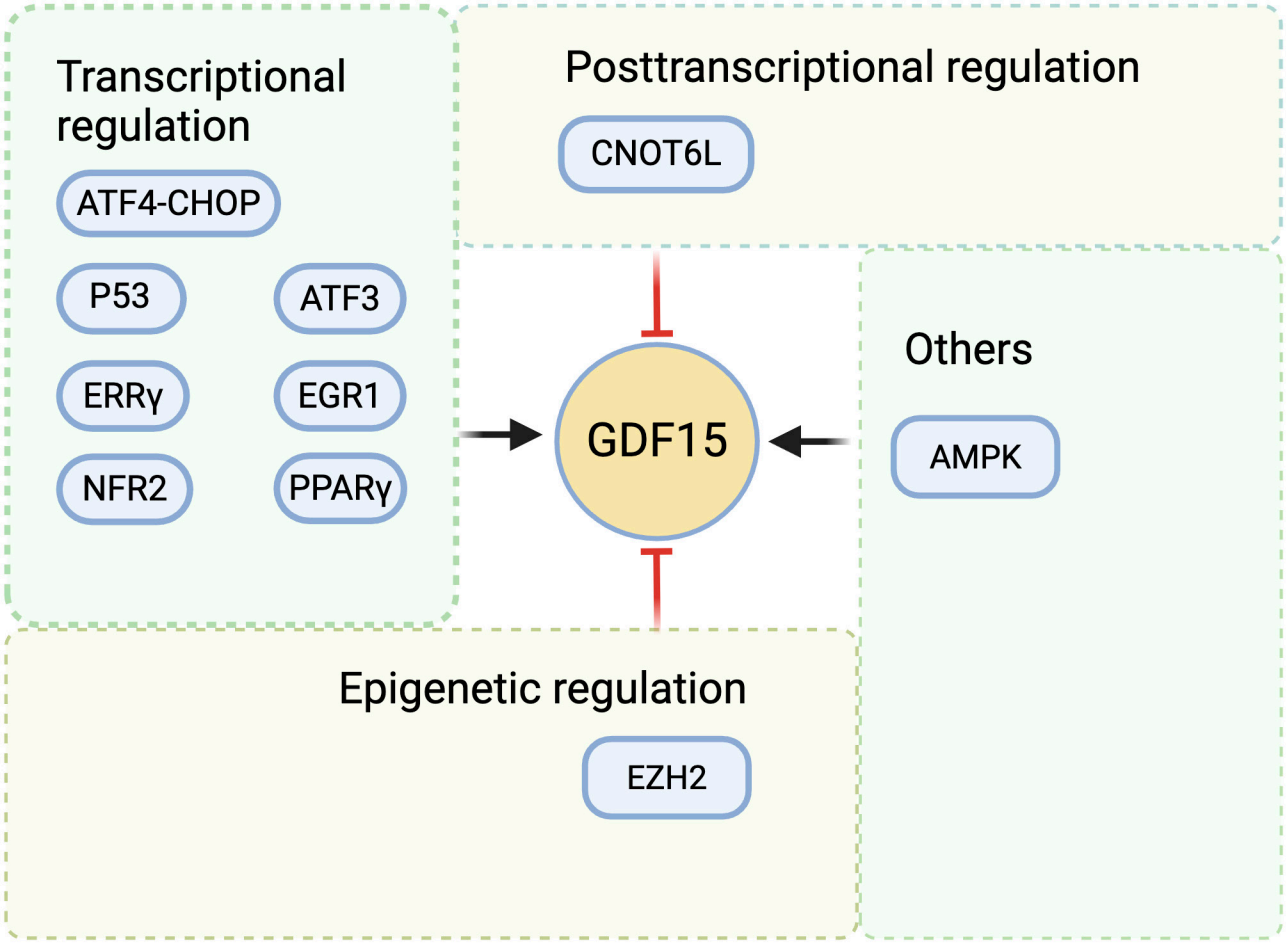
Multilayer regulation of GDF15 expression. The expression of GDF15 is regulated by several factors at multiple levels: (1) transcriptional regulation through stress-responsive factors (ATF4-CHOP complex, p53, ATF3, ERRγ, EGR1, NRF2, and PPARγ) that activate GDF15 in response to diverse cellular stresses; (2) posttranscriptional control mediated by CNOT6L through mRNA decay; (3) epigenetic silencing via EZH2-dependent H3K27me3 modification; and (4) metabolic integration through AMPK signaling. ATF4, activating transcription factor 4; AMPK, AMP-activated protein kinase; CHOP, C/EBP-homologous protein; PPARγ, peroxisome proliferator-activated receptor γ; EGR1, early growth response transcription factor 1; NRF2, nuclear factor erythroid 2-related factor 2; ERRγ, estrogen-related receptor γ; EZH2, enhancer of zeste homolog 2; CNOT6L, CCR4-NOT transcription complex subunit 6.

As a biomarker of cellular stress, GDF15 is up-regulated rapidly and chronically through various mechanisms in the presence of stress stimuli induced by nutritional imbalances, alcohol, hypoxia, endoplasmic reticulum (ER) stress, and amino acid starvation [2,6,25]. These stress stimuli activate key regulators of the integrated stress response (ISR), where eIF2α phosphorylation serves as the central regulatory node. This posttranslational modification enhances activating transcription factor 4 (ATF4) translation, which subsequently dimerizes with C/EBP homologous protein (CHOP) to form a transcriptionally active complex that directly modulates GDF15 promoter activity [19,26,27]. Additionally, stress-responsive transcription factors such as p53 and early growth response transcription factor 1 (EGR1) also serve as critical regulators of GDF15 expression [28–30]. Structurally, the GDF15 promoter contains 2 functional p53-binding sites that enable the cellular stress response [29]. Notably, in head and neck cancer cells, GDF15 forms a positive feedback loop with EGR1. EGR1 directly activates GDF15 transcription through promoter binding, while GDF15 reciprocally up-regulates EGR1 expression via autocrine signaling [31]. Under oxidative stress conditions, NRF2 serves as a master regulator that transcriptionally controls GDF15 expression through direct promoter interaction [32]. In a cysteine deficiency model, rapid glutathione depletion markedly up-regulates NRF2 in the liver and muscle, triggering oxidative stress. This, cooperatively with the ISR, induces the up-regulation of FGF21 and GDF15 [33]. A study on liver injury demonstrated that hepatic estrogen-related receptor γ (ERRγ) directly binds to and activates the promoter region of *GDF15*, thereby regulating *GDF15* expression during hepatic damage [34]. During metabolic liver disease progression, particularly at the critical transition stage from metabolic dysfunction-associated fatty liver disease (MASLD) to MASH, hepatic stress signaling activates transcription factors TFEB and DDIT3 to cooperatively regulate *Gdf15* transcription [35]. Animal studies demonstrate that dual knockdown of TFEB and DDIT3 in hepatocytes markedly reduces hepatic *Gdf15* mRNA expression and circulating GDF15 protein levels in choline-deficient, L-amino acid-defined (CDAA) diet-induced mouse models [35]. Adenosine monophosphate (AMP)-activated protein kinase (AMPK), a key regulator of cellular energy balance, influences GDF15 expression through mechanisms linked to energy stress [36]. Activation of AMPK, either via increased AMP/ATP (adenosine triphosphate) ratios or pharmacological agents, has been shown to up-regulate GDF15 levels in liver and serum [37]. This effect is partially dependent on the AMPK β1-isoform and appears independent of the unfolded protein response (UPR) [36]. This study emphasizes the role of AMPK in stress adaptation and metabolic regulation. Further research is needed to explore their relevance and identify the key tissues contributing to elevated circulating levels of GDF15. Another study indicated that GDF15 regulates AMPK activation. GDF15 deficiency leads to activation of the TGF-β1/SMAD3 signaling pathway, thereby reducing AMPK phosphorylation via protein phosphatase 2A (PP2A) [38]. Furthermore, studies have found a positive feedback regulation between AMPK and GDF15. Metformin up-regulates GDF15 expression via the AMPK/ATF3 pathway. The up-regulated GDF15, in turn, enables AMPK to achieve full activation under metformin treatment [39]. In addition, peroxisome proliferator-activated receptor γ (PPARγ) can directly regulate GDF15 expression at the transcriptional level, whereas PPARβ/δ-mediated increase in GDF15 requires the involvement of AMPK and p53 [37,40]. Epigenetic regulation involves the methyltransferase EZH2, which represses GDF15 transcription by catalyzing trimethylation of histone H3 lysine 27 (H3K27me3) and forming inhibitory chromatin structures at the promoter region [41]. In terms of posttranscriptional modification, CNOT6L, the catalytic subunit of the CCR4-NOT deadenylase complex, can lead to increased degradation of GDF15 transcripts by targeting the 3′ poly(A) tail of GDF15 mRNA [42]. Moreover, in mitochondrial myopathy progression, both GDF15 and FGF21 are markedly up-regulated by the integrated mitochondrial stress response (ISRmt), serving as molecular signatures of this disease [43]. Further highlighting the link between cellular energy stress and GDF15 induction, a study in mice with liver-specific oxidative phosphorylation (OxPhos) dysfunction indicated that activation of the mitochondrial stress response could lead to secretion of GDF15 [44]. Collectively, in Fig. 2, we integrate the multilayered regulatory network governing GDF15 expression under cellular stress conditions, including core mechanisms of transcriptional activation, signaling pathway crosstalk, epigenetic modification, and posttranscriptional regulation.

## The GDF15 Receptor, GFRAL

In 2017, 4 independent groups reported the discovery and characterization of the receptor for GDF15, namely, GDNF-family receptor α-like (GFRAL) [45–48]. GFRAL is a type I transmembrane protein with a molecular weight of approximately 44 kDa [45–48]. It consists of 395 amino acid residues, with a signal peptide at the N terminus and a glycosylphosphatidylinositol (GPI) anchor that attaches it to the cell membrane. The receptor has a short intracellular domain that lacks the ability to conduct signals intracellularly, but it possesses a unique hydrophobic pocket that mediates the interaction between GFRAL and its specific ligand, GDF15 [45–48]. GFRAL is selectively expressed in neuronal cells in the area postrema (AP) and the nucleus of the solitary tract (NST) in brainstem [45–49]. The interaction between GFRAL and GDF15 plays a crucial role in regulating energy metabolism and appetite. Upon binding to GDF15, GFRAL, with the assistance of the tyrosine kinase-associated receptor RET, activates the downstream signaling pathways, leading to multiple biological effects. A study demonstrated that genetic ablation of GFRAL completely abrogated the anorexigenic and weight-reducing effects of GDF15 in diet-induced obese mice. These findings establish GFRAL as the essential receptor mediating the role of GDF15 in the central regulation of energy homeostasis [45–48]. The molecular structure of GFRAL, its specific expression in the brainstem, and the key signaling pathway that mediates the central metabolic role of this ligand/receptor interaction were illustrated in Fig. 1.

## GDF15 and Cardiometabolic Disease

### Obesity

GDF15 plays a well-established role in appetite regulation via the GFRAL/RET pathway in murine models. Overexpression of GDF15 leads to lower energy intake and resistance to obesity, while its down-regulation is associated with increased body fat [45]. Wild-type mice show a decline in food consumption when administered GDF15, an effect absent in GFRAL-deficient mice, reinforcing the necessity of this receptor for GDF15-mediated weight loss [45–48]. However, its broader impact on metabolism and energy expenditure (EE) remains controversial due to conflicting experimental results. These discrepancies are likely influenced by variations in study design, including differences in GDF15 plasma concentrations and methods of administration, whether through recombinant injections or endogenous overexpression. Studies have shown that administering recombinant GDF15 to obese mice leads to weight loss, smaller fat droplets, and reduced liver fat accumulation [47,50]. Additionally, enhanced activity of oxidative and lipolytic enzymes suggests a metabolic shift toward lipid oxidation [18,51]. Calorimetry readings indicate enhanced EE, but the lack of body mass-matched controls complicates interpretations, as body mass itself affects metabolic rate. Investigations using *Gfral*-deficient mice have yielded conflicting results, with some showing increased adiposity on a high-fat diet (HFD), while others report no change [45,47,52]. Interestingly, sex-based differences have emerged, with female *Gdf15* knockout mice displaying higher caloric intake and lower EE, while male counterparts remain unaffected [53].

Early studies utilizing pair-feeding protocols—wherein food intake is matched across groups—suggest that GDF15 influences weight primarily through appetite suppression rather than by directly raising EE [48,54]. However, newer findings challenge this assumption, showing that mice treated with GDF15 exhibit sustained weight loss beyond what pair-fed controls experience, suggesting additional effects on EE [18,46]. A proposed mechanism involves futile ATP consumption via partial uncoupling of the SERCA pump in skeletal muscle, mediated by GDF15-induced sympathetic signaling [50]. Nonetheless, other studies involving tumor-bearing mice with persistently elevated endogenous GDF15 suggest no change in EE [55], highlighting the potential role of fluctuating versus steady-state GDF15 levels in modulating metabolism. GDF15 has also been implicated in mild mitochondrial uncoupling, leading to greater oxygen consumption, carbon dioxide production, and heat generation in mice overexpressing the human *GDF15* gene [50]. In contrast, *Gdf15* knockout mice exhibit diminished metabolic rates, further underscoring its role in energy regulation [45–48]. Recently, an interesting study indicated that dietary cysteine deficiency leads to marked weight loss in obese mice. This lack of cysteine rapidly up-regulates the ISR response in the liver, prompting the release of GDF15 and FGF21, which consequently alters metabolic efficiency [33]. Furthermore, this weight-reducing effect is diminished in *Gdf15* knockout and *Fgf21* knockout mice, confirming the crucial roles of GDF15 and FGF21 in cysteine-deficient diet-induced weight loss [33].

One particularly interesting study explored the interaction between GDF15 and adrenergic signaling. Mice lacking all 3 β-adrenergic receptors (β-less mice) exhibited resistance to GDF15-induced metabolic changes, indicating that the GFRAL–β-adrenergic pathway is instrumental in facilitating EE enhancements [56]. This mechanism likely promotes fatty acid oxidation and calcium cycling in skeletal muscle [50]. However, debate continues regarding the extent to which GDF15 elevates EE independently of feeding behavior. Some research suggests that prolonged GDF15 exposure induces chronic metabolic adaptations, resulting in increased EE beyond what is initially seen with weight loss [50].

Methodological differences likely contribute to the discrepancies observed in studies assessing the role of GDF15 in metabolism [26,57]. The source of GDF15—whether through direct administration or pharmacological up-regulation via agents like metformin—appears to influence its metabolic effects [26]. Additionally, research on melanoma-derived GDF15 has shown that it can up-regulate thermogenic and lipolytic genes, leading to improved glucose tolerance and resistance to obesity independent of food intake [58]. GDF15 suppresses adipocyte differentiation through the HOP2/C/EBPα pathway, thereby modulating adipogenic processes [59]. Similarly, GDF15 administration in wild-type mice up-regulates lipid metabolism-related enzymes, an effect blunted in mice lacking adipose triglyceride lipase (ATGL) or following peripheral chemical sympathectomy [60]. This suggests that the influence of GDF15 on metabolism is partially mediated by sympathetic nervous system activity. GDF15 serves as a molecular mediator linking peripheral fat metabolism with anxiety-like behaviors. Psychological stress elevates β-adrenergic signaling in mice, which enhances lipolysis in white adipose tissue and subsequently stimulates M2-like macrophages within adipose depots to secrete GDF15. This adipokine then activates its central receptor, GFRAL, thereby triggering anxiety-like behaviors—a mechanistic cascade that illuminates the intricate interplay between metabolic processes and neural activity [60].

Furthermore, clinical studies have revealed that bitter-tasting drugs increase endogenous GDF15 expression in obese patients via bitter taste receptors (TAS2Rs) in the intestinal epithelium, suggesting that TAS2Rs may represent novel therapeutic targets for combination therapy in obesity [61]. In one study, gastric bypass surgery exclusively increased serum GDF15 levels in obese patients without metabolic syndrome. However, no correlation was observed between the extent of weight reduction and GDF15 elevation. Consequently, the authors propose that GDF15 likely functions as a biomarker reflecting the severity of metabolic dysregulation rather than directly mediating the surgical weight loss mechanism [62].

These findings highlight the complexity of the metabolic role of GDF15. While its anorectic effects are well established, the extent to which it independently enhances EE remains debated. Given the conflicting evidence, it is plausible that the impact of GDF15 on EE is context dependent, requiring pharmacologically relevant plasma concentrations and prolonged exposure to trigger sustained metabolic changes. The interactions between GDF15 and lipid metabolism suggest that it may hold therapeutic potential for obesity and relevant metabolic diseases, although further investigations are needed to clarify its precise mechanisms and long-term effects (and side effects). Future studies should focus on elucidating the precise pathways through which GDF15 modulates EE and determining whether or not its long-term application can be harnessed for therapeutic purposes in obesity.

### GDF15 and type 2 diabetes mellitus

GDF15 has emerged as a promising therapeutic candidate for type 2 diabetes mellitus (T2DM), largely due to its impact on glucose metabolism, insulin sensitivity, and weight regulation. However, its role remains complex, especially in relation to its potential to improve glucose homeostasis independently of weight loss. Research has shown that GDF15 treatment in animal models (including rodents, shrews, and monkeys) results in reduced body weight and lower blood glucose levels [9,47,51,63,64]. Experiments where food intake was controlled (pair feeding) revealed that glucose-lowering effects of GDF15 were not entirely attributed to weight loss [48], suggesting that GDF15 may directly influence the regulation of glucose metabolism. Despite these findings, larger epidemiological studies have not consistently confirmed a clear association between GDF15 levels and the development or persistence of T2DM, possibly due to confounding factors like body weight, age, and medication use, where, for example, metformin is known to increase GDF15 levels [11,65,66].

One key aspect of the action of GDF15 is its ability to improve insulin sensitivity and glucose tolerance. However, GDF15 is not associated with the insulin-sensitizing effects induced by long-term exercise [67]. Several studies have indicated that GDF15 enhances insulin action, even in the absence of marked weight loss. For example, euglycemic-hyperinsulinemic clamp studies in murine models demonstrated that GDF15 administration enhanced insulin sensitivity independent of body weight alterations [56]. This effect was particularly pronounced in adipose tissue and the liver, both critical organs in glucose metabolism [56]. These findings support the notion that GDF15 may exert its effects through organs other than muscle, challenging the traditional understanding that insulin sensitivity is mainly influenced by muscle glucose uptake. Moreover, a direct action of GDF15 on the liver to reduce glucose production has been linked to changes in the TGF-β1/SMAD3 signaling pathway, which in turn reduces the expression of gluconeogenic enzymes like PEPCK and G6Pase [38]. This mechanism highlights how GDF15 might regulate blood glucose levels by modulating liver function. These effects are further strengthened by the discovery that hepatic-specific restoring GDF15 expression of *Gdf15*-deficient mice led to improvements in insulin sensitivity without major changes in body weight [68]. In mice lacking the GFRAL receptor, or those with pharmacological (propranolol) or genetic (β1/β2 receptor knockout) disruption of adrenergic signaling, the impact of GDF15 on insulin sensitivity and glucose tolerance was abolished, highlighting the significance of the GFRAL/β-adrenergic signaling axis in GDF15-mediated insulin sensitization. While the anorectic effect of GDF15 is strictly GFRAL dependent, it remains β-adrenergic independent, revealing pathway divergence between metabolic and feeding regulation [50,56].

In addition to its peripheral effects, recent research has pointed to the role of GDF15 in pancreatic function. GDF15 levels were reduced in the islets of T1DM patients, whereas no such decrease was observed in T2DM patients [69]. Studies have shown that recombinant GDF15 enhances glucose-stimulated insulin secretion both in vitro and in vivo [70,71]. GDF15 administration promotes β-cell proliferation and survival [72,73].

Overall, while GDF15 exhibits therapeutic potential for treatment of T2DM, particularly due to its ability to enhance insulin sensitivity and glucose tolerance independently of weight loss, much remains to be understood. More in vivo studies are necessary to elucidate its role in glucose homeostasis under physiological conditions and to determine how these mechanisms could be harnessed for T2DM treatment in humans.

### GDF15 and cardiovascular disease

In healthy human cardiovascular systems, GDF15 maintains basal expression but shows marked elevation during cardiovascular pathologies such as pressure overload, heart failure, ischemia–reperfusion injury, and atherosclerosis [5,17]. Although the specific cell types responsible for this elevation of GDF15 level remain elusive, heart-resident macrophages are likely involved and this involvement has been demonstrated to influence the progression of cardiovascular diseases [74]. This is particularly evident in atherosclerosis, where plaque-associated macrophages demonstrate notable GDF15 induction [75]. However, the role of GDF15, as for other TGF family members, in atherosclerosis, remains contradictory. Some studies suggest that GDF15 may promote atherosclerosis development, while others indicate that it could be protective. For example, GDF15 deficiency in ApoE and LDLR knockout mice leads to reduced artery stenosis and decreased macrophage inflammation in plaques [75,76], potentially suggesting that the protein has a proatherogenic effect. On the other hand, other research shows that GDF15 could exert protective effects in atherosclerosis, as in models where GDF15 deficiency increases macrophage accumulation and destabilizes plaques [77,78]. Our recent study revealed that GDF15 deficiency did not influence atherosclerosis progression in either male or female mice injected with AAV8-PCSK9-D377Y [79]. These contradictory findings could be due to variations in experimental conditions, such as mouse strain or environmental factors, combined with the predominant focus of these studies on specific plaque locations without systematically assessing atherosclerotic lesion severity throughout the entire vascular system [80]. In myocardial infarction and ischemia–reperfusion injury, GDF15 exerts a cardioprotective effect by reducing cell death and limiting tissue damage. GDF15 deficiency in mouse models of heart attack or ischemia leads to larger infarcts and greater cardiomyocyte apoptosis [17,81,82]. Conversely, recombinant GDF15 protects cardiomyocytes from ischemia-induced apoptosis [83]. In addition, GDF15 has been shown to inhibit thrombus formation by preventing platelet activation, further supporting its protective role in cardiovascular events. Regarding cardiac hypertrophy, GDF15 levels correlate with increased left ventricular wall thickness in hypertensive patients [84], but studies in mice suggest that GDF15 may prevent or reduce hypertrophy and heart failure. Recombinant GDF15 treatment or overexpression in the heart has been shown to reduce hypertrophy and ventricular dilation [85], likely through pathways including SMAD signaling, AKT, and extracellular signal–regulated kinase 1/2 (ERK1/2) inhibition [17,81]. In diabetic cardiomyopathy, GDF15 can alleviate diastolic dysfunction in T2D mice by reducing myocardial inflammation [86]. Moreover, diabetic patients with higher serum levels of GDF15 have a higher risk of developing peripheral artery disease [87].

Overall, GDF15 appears to have a complex role in cardiovascular disease, acting as both a potential disease amplifier and protector, with further studies required to clarify its mechanisms and physiological significance.

### GDF15 and MASLD/MASH

GDF15 has emerged as a notable biomarker in metabolic dysfunction-associated fatty liver disease/metabolic dysfunction-associated steatohepatitis (MASLD/MASH) through cross-species investigations. Elevated GDF15 levels have been observed in the liver of obese mice and humans with hepatic steatosis [88,89]. In human cohorts, circulating GDF15 correlates with MASLD severity [15,18,90]. This is further supported by a study of over 400 liver biopsy samples, where GDF15 levels correlated with worsening fibrosis, steatosis, and inflammatory activity [91]. These findings indicate that GDF15 not only functions as a biomarker of disease severity but also may have utility as a prognostic indicator for advanced liver fibrosis. During MASLD development, the elevated levels of GDF15 primarily originate from the liver, especially from hepatocytes [27,88]. How GDF15 precisely exerts its biological actions remains incompletely understood and a focus of ongoing research. Rodent models suggest that elevated GDF15 may represent a compensatory response to hepatic stress, potentially mitigating MASLD/MASH progression by modulating inflammatory and fibrotic pathways. Conversely, genetic ablation of GDF15 in mice exacerbates hepatic inflammation and fibrosis, while exogenous GDF15 administration attenuates these pathologies [18,88].

In animal models, both recombinant GDF15 and its overexpression have been shown to reduce hepatic steatosis, inflammation, and fibrosis, independent of weight loss [27,88]. This suggests that GDF15 may influence liver pathology through mechanisms beyond its known appetite-suppressing effects. Notably, GDF15 appears to enhance fatty acid oxidation and protects against mitochondrial dysfunction in liver [18], indicating its potential in mitigating liver injury associated with MASH.

### Others

Cachexia represents a complex metabolic disorder involving weight loss, reduced appetite, and the progressive wasting of muscle and loss of fat stores [92]. Cachexia severely affects patients’ quality of life and reduces their survival rates. While the pathogenesis of cancer cachexia involves metabolic, immune, and endocrine disruptions, GDF15 has emerged as one of the central players in this pathology [93]. Elevated levels of GDF15 occur in various cancers, including prostate, colon, and pancreatic cancers [54,94,95], and is closely related to weight loss and a decrease in body mass index (BMI) [96,97]. GDF15 primarily drives cachexia by inducing anorexia, leading to reduced nutritional intake [98,99]. Classified as a myokine, GDF15 shows an inverse relationship between its circulating levels and muscle mass in cancer cachexia [100]. Current research suggests that the reduction in muscle mass observed in cachectic individuals is not solely due to anorexia. GDF15 has been shown to boost the production of proteins like MuRF1 and MAFbx/atrogin-1, which are key drivers of muscle breakdown [101,102]. GDF15 may also trigger muscle atrophy possibly by directly interacting with specific receptors to induce muscle loss such as MAP3K11 [101]. GDF15 might indirectly contribute to muscle wasting by promoting lipid oxidation and fat catabolism, which, in turn, exacerbates the metabolic imbalance [103]. Studies in murine models have shown that high levels of GDF15 correlate with marked reductions in fat depots, particularly in visceral fat. This effect is mediated through both SMAD and non-SMAD signaling pathways, which are involved in the regulation of lipid metabolism [104]. GDF15 promotes the development of bone loss while suppressing osteoblast differentiation and increasing osteoclast differentiation [105]. Additionally, GDF15 may interact with other members of the TGF-β superfamily, such as GDF11, which is known for its role in regulating muscle growth [106]. Monoclonal antibody therapies targeting GDF15 are currently being explored in clinical trials, offering new hope for the treatment of cancer cachexia.

GDF15 is also a key contributor to the development of cardiac cachexia. Levels of GDF15 are increased in patients with cardiac cachexia and are closely associated with myocardial fibrosis, impaired cardiac function, and the severity of cachexia [107]. Animal studies have shown that monoclonal antibodies targeting GDF15 can prevent weight loss and the onset of cachexia while also slowing the deterioration of cardiac function [108]. These findings offer exciting new hope for the treatment of cardiac cachexia. Further investigations are necessary to optimize these therapies and clarify the full spectrum of the involvement of GDF15 in cachexia and other cancer-related complications.

Although primarily linked to cancer cachexia, recent studies have identified GDF15 as a key regulator of muscle deterioration in conditions like sarcopenia [109–111]. Emerging evidence demonstrate that GDF15 dysregulates proteostasis by suppressing protein synthesis and activating ubiquitin–proteasome-dependent protein degradation pathways. Neutralizing GDF15 may be a promising therapy for enhancing muscle function and physical performance in mice with mitochondrial myopathies [112].

GDF15 is expressed at low levels in healthy young individuals but markedly increases with age, making it one of the most up-regulated proteins during aging [113,114]. GDF15 has been specifically identified as a constituent of the senescence-associated secretory phenotype (SASP) [115]. A recent study demonstrated that the regulatory effects of perivascular adipose tissue (PVAT) on vascular aging are partially mediated by GDF15 [116]. Hepatocyte senescence with elevated expression of the SASP factor GDF15 was closely associated with alcoholic hepatitis [117]. The role of GDF15 in aging is complex [118]. Further research is needed to elucidate its precise mechanisms and therapeutic potential in aging-related conditions.

## Interactions of GDF15 with Other Endogenous Metabolic Factors

### FGF21

FGF21 and GDF15, both recognized as mitochondrial stress response factors [20,119], are co-regulated through shared pathways such as the ISR and play crucial roles in maintaining systemic metabolic homeostasis [6,120,121]. These factors exhibit parallel expression patterns in various pathological conditions, including obesity, MASLD, and mitochondrial disorders [122,123]. They act complementarily to preserve metabolic stability: GDF15 primarily suppresses energy intake, whereas FGF21 enhances insulin sensitivity and promotes EE [42,44]. Bidirectional compensatory up-regulation is observed in genetic deficiency models—*Fgf21*^−/−^ mice display elevated plasma GDF15 concentrations, while Gdf15^−/−^ mice exhibit increased circulating FGF21 levels and hepatic *Fgf21* mRNA expression [124]. In GFRAL-knockout obese female mice, the weight-reducing effect of FGF21 is impaired (likely due to increased food intake), yet its glucose-improving actions remain intact [125]. FGF21 overexpression does not alter circulating GDF15 levels, and neither factor modulates the central expression of the other’s receptor, indicating largely independent signaling pathways [125]. In MASLD, GDF15 supplementation induces hepatic FGF21 expression, and their crosstalk contributes synergistically to therapeutic efficacy [126].

Future investigations should focus on unraveling both the interplay of GDF15 and FGF21 and the biological mechanisms driving their functional outcomes.

### GLP-1

GLP-1R agonists like liraglutide promote weight loss [127]. Although GLP-1R and GDF15 receptor (GFRAL) expression overlap in hindbrain regions, GDF15 does not activate GLP-1R neurons [48,128,129], and the effects of each pathway appear distinct, as demonstrated by studies in knockout mice [128]. Liraglutide also did not affect GDF15 levels in obese humans [130]. However, combined treatment with GDF15 and GLP-1R agonists synergistically enhances weight loss in mice. Dual agonists (e.g., QL1005) show promise for greater weight reduction in preclinical studies [131].

### Leptin

Leptin functions as a key hormone in regulation of EE by suppressing appetite [132]. Previously, it was believed that the anorectic effects of GDF15 and leptin mediated appetite suppression via functionally distinct pathways, as leptin maintained its ability to inhibit food intake in *Gfral*-knockout mice [48], and GDF15 could induce hypophagia and weight loss in ob/ob (leptin-deficient) mice [54]. However, a study revealed a synergistic effect between GDF15 and leptin, suggesting that GDF15 and leptin can synergistically induce weight loss, possibly due to the association between GDF15 and leptin signaling pathways within AP and NST [133]. This research advanced our understanding of how GDF15 and leptin modulate energy balance.

### Glucocorticoids

The biological relationship between GDF15 and glucocorticoids is bidirectional. Conditions of glucocorticoid deficiency, such as in primary adrenal insufficiency (PAI), are associated with increased GDF15 concentrations. This elevation is reversed upon glucocorticoid replacement, demonstrating that glucocorticoids exert a regulatory effect on GDF15 levels [134]. Exogenous administration of GDF15 in mice results in the activation of Crh neurons, leading to an increase in corticosterone levels, which is indicative of hypothalamic–pituitary–adrenal (HPA) axis activation [135]. While the studies provide valuable insights into GDF15 and glucocorticoid interactions, several questions remain unanswered. For instance, the exact mechanisms underlying GDF15’s activation of the HPA axis and its regulation by glucocorticoids are still largely unknown.

## Dietary Influence

### Ketogenic diet

The ketogenic diet is characterized by its high-fat, adequate-protein, and very-low-carbohydrate (or even no-carbohydrate) composition, the diet being designed to induce a state of metabolic ketosis similar to fasting [136]. Emerging evidence indicates that consumption of a ketogenic diet in mice, pigs, and humans triggers a marked elevation in circulating GDF15 levels, concomitant with suppressed caloric intake and weight reduction [137]. Notably, among prevalent dietary strategies for obesity management—such as the Mediterranean diet, low-fat diet, high-protein diet, and low-glycemic-index diet—the ketogenic diet uniquely stimulates GDF15 expression [137]. The study also corroborated that ketogenic diet-induced GDF15 originates from the activation of hepatic PPARγ, which governs GDF15 transcription and production [137].

Traditionally, the weight loss associated with ketogenic diet has been attributed to its ability to induce ketosis, which shifts the body’s metabolism away from glucose and toward fat as the primary energy source [136]. However, the role of GDF15 in this process adds a new layer of complexity and opens avenues for further research. The therapeutic promise of the ketogenic diet in diverse pathologies, including cancers, epilepsy, and aging, is being evaluated in ongoing clinical studies (ClinicalTrials.gov database). When conducting clinical trials involving ketogenic diet, it may be crucial to closely monitor GDF15 levels to ensure patient safety and the validity of the trials.

### Fatty acids

Fatty acids have been shown to increase serum levels of GDF15 in a dose-dependent manner [16,138]. Fatty acids exert their anorectic effects primarily through the GDF15–GFRAL signaling axis [16]. Interestingly, certain fatty acids appear to boost GDF15 levels in specific organs. Medium-chain fatty acids mainly raise *Gdf15* mRNA in the liver and small intestine [138]. The kidney is identified as the primary tissue responsible for this increase in GDF15 expression, with elevated levels observed within the cortex and outer medulla [16]. The identification of the kidney as a major source of GDF15 in response to fatty acids opens up new avenues for research into the potential role of kidney-derived cytokines in regulating food intake and body composition. This tissue-specific response could be linked to the way medium-chain fatty acids are absorbed directly into the bloodstream through the hepatic portal vein, unlike other fatty acids, which travel via the lymphatic system before entering circulation through the vena cava. The underlying mechanisms are still not fully understood.

## Pharmacological Induction of GDF15

**Fig. 3.**
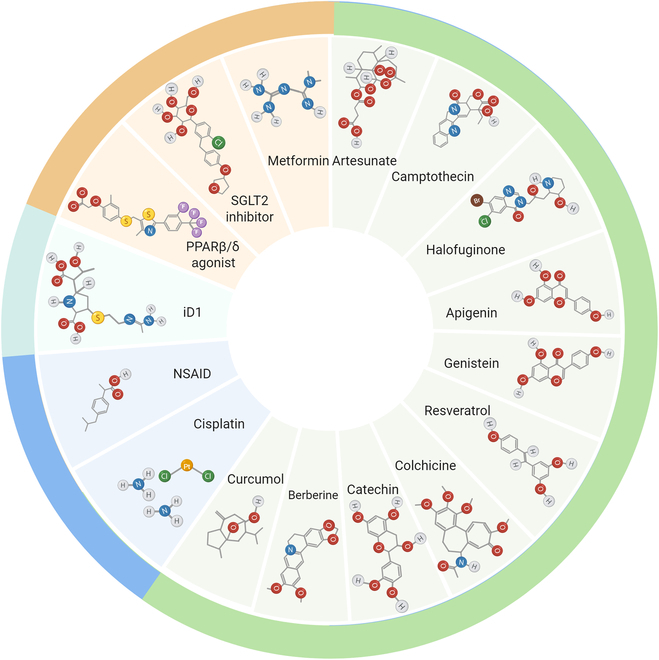
Pharmacological Inducers of GDF15. GDF15, a pleiotropic cytokine, is pharmacologically induced by diverse pharmaceutical agents that modulate its expression and downstream metabolic effects. The glucose-lowering drug metformin elevates circulating GDF15 levels, thereby suppressing feeding activity to regulate body weight. Similarly, SGLT2 inhibitors and PPARβ/δ agonists enhance GDF15 expression, contributing to metabolic benefits beyond glycemic control. Among small-molecule natural compounds, artesunate reduces obesity in a GDF15-dependent manner, while CPT alleviates adiposity via activation of the GDF15–GFRAL axis. Halofuginone exhibits dual mechanisms: It up-regulates GDF15 to suppress appetite and synergizes with FGF21 to promote EE, collectively mitigating obesity. Curcumol induces GDF15 production, consequently ameliorating obesity by activating ER stress. Berberine ameliorates obesity by inducing GDF15 secretion from brown adipocytes. Additionally, natural compounds such as catechin, apigenin, genistein, resveratrol, and colchicine exert anticancer effects by up-regulating the GDF15 levels. Anti-inflammatory agents like NSAIDs induce GDF15 gene expression independently of COX inhibition, whereas chemotherapeutic drugs (e.g., cisplatin and doxorubicin) elevate GDF15 levels, potentially exacerbating chemotherapy-induced anorexia and weight loss through GDF15-mediated pathways. CONT6L promotes degradation of GDF15 mRNA by targeting its 3′ poly(A) tail, while CONT6L inhibitor (iD1) enhances GDF15 mRNA stability, thereby elevating circulating GDF15 levels. SGLT2, sodium-dependent glucose transporters 2; PPARβ/δ, peroxisome proliferator-activated receptor β/δ; NSAIDs, nonsteroidal anti-inflammatory drugs.

Several clinically approved drugs or candidate compounds have been unexpectedly found to robustly elevate circulating GDF15 levels, suggesting that part of their therapeutic effects may be mediated through this pleiotropic hormone (Fig. 3). The analysis of these pharmacological inducers serves 3 critical purposes: First, it may reveal common pathways of GDF15 regulation that could be targeted therapeutically. Second, it provides clinical validation of GDF15’s importance in human metabolic physiology through “natural experiments” of drug effects. Third, understanding these drug–GDF15 relationships may explain certain therapeutic benefits (and possibly side effects) of these widely used medications. This section will thus systematically evaluate the evidence for pharmacological induction of GDF15 and its potential clinical implications for cardiometabolic disease management.

### Metformin

Metformin, a widely prescribed drug for the treatment of T2D, has been in the clinical spotlight lately for its various potential therapeutic effects beyond blood glucose control [139,140]. Recently, the relationship between metformin and GDF15 has garnered considerable interest. Initial investigations involving serum analysis from over 8,000 dysglycemic participants demonstrated a substantial rise in GDF15 levels following metformin treatment independently of other factors including age, BMI, and hemoglobin A1c (HbA1c) [141]. However, the underlying mechanisms of increased GDF15 in mediating the benefits of metformin remain unknown. In 2019, 2 pivotal studies revealed that metformin induces the expression and secretion of GDF15, leading to increased levels of GDF15 in the blood [26,57]. These elevated GDF15 levels combine to GFRAL, suppressing appetite, thereby facilitating weight loss. Knocking out the GDF15 or GFRAL gene in mice abolished the anorexic and weight-reducing effects of metformin [26]. Data from clinical, cellular, organoid, and mouse models identified the distal small intestine and colon as the primary sources of GDF15 in response to metformin treatment [26]. Another study indicated that metformin directly stimulates the secretion of GDF15 in primary mouse hepatocytes [57]. Subsequent research revealed that the kidney is the key organ responsible for the anti-obesity effects of metformin [8]. This discovery uncovers a previously unknown function of the kidney in regulating energy balance. The study also analyzed GDF15 levels in over 300 human kidney biopsy samples and found a correlation between kidney GDF15 levels and its blood levels, supporting their findings. However, Klein et al.’s [142] research revealed that, despite inducing an elevation in serum GDF15 levels, metformin promotes weight loss via GDF15–GFRAL-independent mechanisms in mice and humans. These variations may be attributed to the relatively short treatment duration with metformin.

The underlying mechanism of how metformin induces GDF15 expression remains largely unexplored. Metformin stimulated ISR and increased ATF4-CHOP expression [8,26,57]. *Gdf15* mRNA levels were increased in parallel with the fold elevation of eIF2α phosphorylation, CHOP, and ATF4 in response to metformin [8,26,57]. Genetic deletion of *Atf4* or *Chop* eliminated the metformin-induced secretion of GDF15 [26]. Further research is necessary to elucidate the role of eIF2α as well as its downstream transcriptional factors ATF4 and CHOP. Additionally, metformin activates AMPK and increases GDF15 levels in hepatocytes and myotubes, with GDF15 being essential for sustaining full AMPK activation independently of the central nervous system [39]. In mice, metformin’s glucose-lowering effects and AMPK activation in the liver and skeletal muscle are dependent on GDF15, as these effects are absent in *Gdf15*^−/−^ mice [39].

These studies have illuminated the complex interplay between metformin and GDF15. The discovery of the role of GDF15 in the anti-obesity effects of metformin offers new possibilities for targeted therapies to address the global obesity epidemic.

### Artesunate

Artesunate, a derivative of artemisinin primarily used for treating malaria, has garnered attention in recent years for its potential anticancer and anti-inflammatory properties [143,144]. Recent research revealed that treatment with artesunate exhibited marked weight loss effects in diet-induced obesity (DIO) mice and Cynomolgus macaques with spontaneous obesity [145]. This weight loss effect is attributed to the suppression of energy intake [145]. Additionally, the monkeys also exhibited improved insulin sensitivity and lower blood lipids, without inducing adverse effects such as nausea and malaise [145]. Furthermore, the anti-obesity benefits of artesunate were shown to require GDF15 signaling, as gene deletion of GFRAL or cell-type-specific silencing of GDF15 abolished its metabolic effects [145]. The study identified the liver as a key source of GDF15 [145]. The mechanism through which artesunate induces GDF15 production involved the activation of the ISR with CHOP as a primary mediator [145].

Future studies are likely to focus on refining the dosage and administration protocols of artesunate to maximize its efficacy while minimizing potential side effects. Clinical trials in humans will be crucial to validate the findings from animal studies and to establish artesunate as a safe and effective treatment option for obesity.

### Halofuginone

Halofuginone, a U.S. Food and Drug Administration-approved drug for scleroderma and protozoal infections [146], works through inhibition of aminoacyl-tRNA synthetases, particularly glutamyl-prolyl-tRNA synthetase (EPRS), thereby activating the amino acid starvation response (AAS) and ISR signaling pathways [147,148]. Recently, we found that halofuginone promotes weight loss in DIO mice and pigs by suppressing food intake and increasing EE [149]. Halofuginone also alleviates insulin resistance and hepatic steatosis [149]. The effects were mediated through the ISR pathway and the up-regulation of GDF15 and FGF21 [149]. Both hormones are necessary for the anti-obesity effects of halofuginone [149]. GDF15 mediates the suppression of food intake, while FGF21 is responsible for increasing EE [149]. Halofuginone shows potential for clinical use with acceptable safety at low doses [149]. However, it induces gastrointestinal adverse effects, such as vomiting and diarrhea, in canine models [149]. Overall, our research highlights halofuginone as a promising candidate for obesity treatment. Further clinical studies are warranted to confirm its efficacy and safety in humans.

### Inhibitor of CNOT6L deadenylase 1

CNOT6/CNOT6L, a central catalytic component of the CCR4-NOT deadenylase complex, is pivotal in mRNA decay by targeting the 3′ poly(A) tail [150]. This tail is crucial for mRNA stability and translation efficiency, and its removal by CNOT6/CNOT6L serves as a key regulatory mechanism for gene expression [151]. The activity of CNOT6/CNOT6L is modulated by various stimulus, such as nutrient levels, highlighting its involvement in metabolic regulation and potential links to metabolic diseases [152,153]. A recent study found that CNOT6L functioned as a critical switch that turned off the expression of GDF15 and FGF21 [42]. Pharmacological inhibition of CNOT6L with iD1 (inhibitor of CNOT6L deadenylase 1) markedly enhanced the stability of *Gdf15* and *Fgf21* mRNAs, leading to elevated circulating levels of GDF15 and FGF21 proteins [42]. This, in turn, stimulated the brain and peripheral tissues, resulting in reduced food intake, increased EE, and weight loss [42]. It is worth noting that the study also revealed a delay between the increase in hepatic *Gdf15* mRNA levels and the subsequent increase in serum GDF15 protein levels, suggesting that GDF15 protein accumulates in the liver before being released into the bloodstream [42]. The study provides valuable insights into the posttranscriptional regulation of GDF15 by CNOT6L and highlights the potential of targeting this pathway for the treatment of metabolic disorders. However, further research is needed to fully understand the off-target effects of CNOT6L inhibitors and to explore the therapeutic potential of this approach in clinical settings.

### SGLT2 inhibitors

Sodium-dependent glucose transporters 2 (SGLT2) are high-capacity, low-affinity transporters that are mainly expressed in the kidney and have the primary physiological function of reabsorption of glucose from the glomerular filtrate in the proximal tubules [154]. SGLT2 inhibitors are a promising new class of anti-hyperglycemia drugs that are widely used in patients with type 2 diabetes [155]. Clinical studies have indicated that SGLT2 inhibitors can improve major cardiovascular, renal, and metabolic outcomes irrespective of the presence of diabetes [156]. Large-scale proteomics investigations revealed that treatment with empagliflozin, a selective SGLT2 inhibitor, increased plasma GDF15 levels in patients with T2D [157]. In the post hoc study that included 190 heart failure patients predominantly without diabetes, empagliflozin increased plasma levels of GDF15 [158]. The cardioprotective effects of empagliflozin on heart failure are more pronounced in patients with higher baseline levels of GDF15 [159]. To our knowledge, no medications for heart failure have been proven to alter the levels of GDF15, although the GDF15 level was associated with cardiovascular outcomes. Whether the elevation of GDF15 induced by empagliflozin is associated with its cardiovascular benefits remains unknown and requires further research. However, in another study, canagliflozin treatment was found to lower GDF15 levels in patients with T2D [160]. The decrease in GDF15 levels induced by canagliflozin has been attributed to an improvement in disease status rather than a direct effect of canagliflozin itself.

### PPARβ/δ agonists

PPARβ/δ, a PPAR isoform, serves as a key regulator of glucose and lipid metabolism, as well as inflammatory responses [161]. Upon activation by specific ligands, PPARβ/δ can exert a multitude of beneficial effects, including the reduction of dyslipidemia and hyperglycemia, enhancement of whole-body insulin sensitivity, and prevention of DIO [162]. GDF15 has been identified as a central gene regulated by PPARγ at the transcriptional level, acting as its direct downstream target [30,40]. Previous research has highlighted a pivotal role for GDF15 in facilitating the metabolic actions of PPARβ/δ [37]. Pharmacological activation of PPARβ/δ elevates GDF15 levels, ameliorates glucose intolerance, fatty acid oxidation, ER stress, and inflammation while also activating AMPK in mice fed an HFD [37]. Notably, these effects were abolished when GDF15 was neutralized by antibody administration or in *Gdf15* knockout mice [37]. Evidence suggests that GDF15 induces AMPK activation in both in vitro myotube cultures and skeletal muscle preparations via GFRAL-independent pathways, indicating a GFRAL-independent mechanism [37]. These data collectively establish that GDF15 elevation plays a pivotal role in mediating the antidiabetic effects of PPARβ/δ agonists.

### Curcumol

Curcumol, a bioactive sesquiterpenoid compound isolated from the traditional Chinese medicinal plant *Curcuma longa*, exhibits multiple biological activities including antitumor effects, antioxidant properties, and protective actions against myocardial infarction and MASH [163–165]. Curcumol elevates GDF15 levels in mice by activating the ER stress pathway, subsequently altering food preferences and reducing body weight [166]. Long-term administration of curcumol enhances lipid metabolism and glucose tolerance [166]. Notably, this study demonstrated curcumol’s superior efficacy against obesity compared to metformin [166].

### Berberine

Berberine (BBR), an isoquinoline alkaloid derived from the traditional Chinese medicinal herb *Coptis chinensis*, has demonstrated multiple metabolic benefit including hypoglycemic, lipid-modulating, and anti-obesity effects [167]. Investigations in DIO murine models revealed that BBR administration markedly elevated circulating levels of GDF15 and negative correlation between serum GDF15 concentrations and adiposity metrics suggests that GDF15 may serve as a pivotal mediator of BBR-induced weight management [168]. In vitro analyses demonstrated that BBR stimulates GDF15 secretion in primary brown adipocytes through activation of the ISR pathway [168].

### Chemotherapy drugs

Derived from the Chinese tree *Camptotheca acuminata*, the plant-derived alkaloid camptothecin (CPT) has emerged as a molecule of considerable biomedical interest due to its potent anticancer activity [169]. One study found that CPT effectively addresses obesity in mice through induction of the GDF15–GFRAL pathway [170]. The research team administered low-dose CPT to obese mice and observed a reduction in body weight and food intake compared to control mice, primarily through activation of the ISR in the liver [170]. Notably, CPT-induced GDF15 elevations were obesity dependent, suggesting that the relatively low levels of GDF15 in lean mice were insufficient to suppress food intake and body weight [170].

CPT is known to exhibit a range of adverse effects, including gastrointestinal disturbances, neutropenia, and alopecia [171]. Due to its high toxicity and the side effects associated with high-dose administration required for cancer treatment, its use has been limited [172]. While CPT holds promise as a therapeutic option for obesity, its dosage and toxicity profile must be carefully balanced to ensure safety and effectiveness.

Platinum-based chemotherapy drugs, like cisplatin and nonplatinum chemotherapy agent doxorubicin, have been reported to induce an increase in circulating GDF15 and concomitant weight loss in mice [173]. The increase in GDF15 levels is facilitated by the selective activation of the hepatic IRE1α–XBP1 signaling pathway, which is part of the UPR [173]. Both hepatic IRE1α knockout and IRE1α ribonuclease inhibitors markedly attenuate liver *Gdf15* production and reduce circulating GDF15 concentrations, leading to improved chemotherapy-associated anorexia and body mass reduction [173]. This suggests a broader involvement of GDF15 in the emetic regulation and cachexia associated with different chemotherapy regimens.

Furthermore, GDF15 neutralization resulted in alleviated anorexia and nausea in nonhuman primates [174]. This suggests GDF15 as a potential therapeutic target for mitigating chemotherapy-induced toxicity, enhancing the quality of life for cancer patients undergoing treatment. However, the complex interplay between GDF15 and tumor growth remains unclear, with contradictory findings reported in overexpression and knockdown studies. Further investigation is needed to fully elucidate the role of GDF15 in cancer therapy and to develop effective strategies for its modulation.

### Anti-inflammatory drugs

Nonsteroidal anti-inflammatory drugs (NSAIDs) have long been recognized for their anti-inflammatory properties, primarily attributed to the ability to inhibit cyclooxygenase (COX) enzymes [175]. GDF15, also in context referred to as NAG-1 (nonsteroidal anti-inflammatory drug-activated gene-1), owes its alternate nomenclature to its inducible expression by NSAIDs [176]. NSAIDs, such as indomethacin and ibuprofen, induce GDF15 independently of its COX inhibitory activity [177]. Instead, this process is driven by the activation of the NRF2 (nuclear factor erythroid 2-related factor 2) transcription factor [177]. This suggests that the anti-inflammatory and protective effects of NSAIDs may extend beyond their COX-inhibiting activities, potentially explaining some of the observed clinical benefits of these drugs in conditions not directly related to inflammation or pain. While no marked alterations in endotoxemia progression were observed following GDF15-targeted interventions in their research, this does not rule out GDF15 playing a crucial role under different conditions or aspects [177]. Further exploration of the mechanisms underlying GDF15’s biology and how NSAIDs interact with it is needed.

Colchicine, one of the oldest medications still in clinical use, was historically derived from *Colchicum autumnale* and *Gloriosa superba* [178]. Today, it is widely used to treat inflammatory conditions such as gout, pericarditis, and auto-inflammatory dermatitis [178]. Recent research has clarified how colchicine activates the NRF2 pathway in hepatocytes, thereby inducing the release of hepatokines, with GDF15 being a prominent example [32]. This process identifies a novel communication pathway between hepatic and myeloid cells, where GDF15 plays a crucial role in inhibiting myeloid cell activation and anti-inflammatory effects of colchicine [32]. These findings highlight the promise of GDF15 as an emerging therapeutic candidate for inflammatory pathologies.

### Natural compounds with anticancer activity

The natural compounds introduced in this chapter can markedly up-regulate the expression of GDF15, but it is unknown whether the GDF15-elevating effects contribute to their cardiovascular or metabolic benefits.

Resveratrol is a small-molecule polyphenolic compound produced by various plants including grapes, peanuts, and mulberries [179]. It has attracted appreciable scientific interest due to its potential role in mediating the known cardioprotective effects of red wine. The reported biological activities of resveratrol continue to expand, with numerous direct molecular targets having been identified through in vitro studies. Resveratrol was reported to induce GDF15 expression through p53-mediated transcriptional activation [180,181]. GDF15 was shown to play a pivotal role in resveratrol-induced growth inhibition of various types of cancer cell lines [181,182].

Genistein, a soy-derived isoflavone, demonstrates potent antitumor effects by suppressing proliferation and stimulating apoptosis in colorectal cancer cells while up-regulating the tumor-suppressive protein GDF15 through p53-dependent mechanisms [183].

Apigenin, a kind of dietary flavonoids, exhibits anticancer properties by emerging as particularly effective in suppressing colorectal cancer growth through up-regulation of GDF15 [184]. GDF15 secretion is stimulated when apigenin disrupts the binding of NRF2 to KEAP1, leading to NRF2 degradation [184].

Epidemiological and experimental evidence suggests that catechins, a type of polyphenol found in green tea, have anticancer activity. One study demonstrated that catechins, particularly epicatechin gallate, can activate GDF15 through the ATF3 transcription factor, independent of p53 [185].

## GDF15 as a Therapeutic Target

The pharmacological modulation of GDF15 signaling—through either agonism or antagonism—has emerged as a promising therapeutic strategy for cardiometabolic disorders. While endogenous GDF15 serves as a stress-responsive cytokine with pleiotropic effects on metabolism and tissue protection, exogenous manipulation of this pathway offers distinct clinical advantages. First, GDF15 agonists may replicate or amplify the cytokine’s beneficial metabolic actions, including weight loss, improved insulin sensitivity, and cardioprotection, as demonstrated in preclinical models. Conversely, GDF15 inhibitors could mitigate its potential detrimental effects, such as β-cell apoptosis or cancer-associated cachexia, in specific pathological contexts.

Currently, pharmaceutical companies worldwide have embarked on drug research with GDF15 as a novel therapeutic target, encompassing various fields such as obesity, cancer, and anorexia syndrome. There are various drugs targeting GDF15; however, some of these have ceased development due to challenges faced or termination of the projects. Among the candidate drugs, many are still in early stages of development, including preclinical and clinical trial phases. Specifically, some drugs have progressed to phase 1 and phase 2 clinical trials (Table). These drugs encompass multiple types, including monoclonal antibodies, fusion proteins, small-molecule drugs, and others. We comprehensively analyze the advantages and disadvantages of the GDF15 target, discuss and objectively evaluate the new drug developments based on GDF15, and will provide a scientific basis for the discovery of related innovative drugs.

**Table.** Drugs targeting GDF15–GFRAL pathway

Agent	Indication	Development stage	ClinicalTrials.gov ID/ref.	Company
GDF15 agonists				
MBL949	Obesity	Phase 2	NCT05199090	Novartis Pharmaceuticals
LY3463251	Obesity	Phase 1	NCT03764774	Eli Lilly & Co.
AMG-171	Obesity	Phase 1	NCT04199351	Amgen Inc.
JNJ-9090/CIN109	Obesity/T2DM	Phase 1	NA	Jansenn/CinFina Pharma
YH34160	Obesity	Preclinical	See related links	Yuhan Corporation
NNC0247-0829 (LA-GFD15)	Obesity	Phase 1	NCT04010786	Novo Nordisk
QL1005 (GDF15 /GLP-1 dual agonists)	Obesity	Preclinical	NA	Beijing QL Biopharm Co. Ltd.
CIN-209 (GDF15 /GLP-1 dual agonists)	Obesity	NA	NA	Jansenn/CinFina Pharma
GDF15 inhibitors				
Ponsegromab	Cachexia	Phase 2	NCT05546476	Pfizer Inc.
Heart failure	Phase 2	NCT05492500		
Rilogrotug (AV-380)	Cachexia	Phase 1	NCT04815551	AVEO Pharmaceuticals Inc.
H53E5-8V2	Cachexia	Preclinical	See related links	Kyinno Biotechnology Co. Ltd.
GDF15 mAb (juvenescence)	Muscular atrophy/neoplasms	Preclinical	NA	Juvenescence Ltd.
LBL-049	Cachexia	Preclinical	See related links	Nanjing Leads Biolabs
GFS202A (GDF15 /IL-6 dual antibody)	Cachexia	Preclinical	NA	GenFleet Therapeutics

NA, not available

### GDF15 agonists

#### MBL949

MBL949 is a recombinant human GDF15 dimer that is covalently attached to either 1 or 2 fatty acids capable of binding to albumin, through the use of a short and flexible polyethylene glycol (PEG) linker [186]. In preclinical species, MBL949 demonstrated pronounced weight loss and reduced food intake, attributes that were attributed to its GDF15 bioactivity [63,186]. The prolonged half-life of MBL949 supports biweekly dosing in patients, making it a viable therapeutic option for chronic administration [186]. However, phase 2 clinical trials revealed limited weight loss efficacy despite its acceptable safety profile and predictable pharmacokinetics (PK) supporting biweekly dosing [186]. Gastrointestinal adverse events, such as nausea and vomiting, were frequently reported, potentially limiting its tolerability [186]. The discrepancy between preclinical and clinical outcomes suggests that the GDF15/GFRAL pathway may function differently in humans, or that MBL949’s molecular size and structure hinder its access to relevant brain regions. Future research could explore combining MBL949 with other metabolic regulators, such as GLP-1 agonists, to enhance its therapeutic potential for obesity and related metabolic disorders. Clinical studies on MBL949 for the treatment of cancer anorexia, heart failure, and MASH are also ongoing.

#### LY3463251

LY3463251 is a GDF15 analog designed as a long-acting agonist targeting the GFRAL/RET receptor system [187]. By fusing GDF15 with a modified immunoglobulin G4 (IgG4) Fc domain, the molecule achieves extended circulation time, addressing the limitations associated with the short half-life of native GDF15 [187]. Preclinical studies in rodents and nonhuman primates revealed marked reductions in food intake and body weight, with no adverse effects such as malaise or emesis [187]. In human trials, LY3463251 exhibited a favorable PK profile, supporting once-weekly dosing [187]. However, its weight loss efficacy in overweight and obese individuals was modest, with only a 3% reduction compared to placebo over 12 weeks [187]. The intervention induced dose-related gastrointestinal disturbances (e.g., nausea/vomiting), with no causal connection to its weight-reduction mechanism [187]. Current data suggest that inadequate drug exposure in humans could be the reason for the relatively reduced response. The precise mechanisms through which GDF15 reduces food intake—whether via satiety or nausea/aversion—and promotes weight loss remain unclear, as does their translatability from animal models to humans. These results underscore the complexity of translating preclinical findings to human outcomes, highlighting the need for further research to optimize the therapeutic potential of GDF15 receptor agonism while minimizing adverse effects.

#### AMG-171

AMG-171 is a fusion protein that combines the GDF15 subunit with an Fc fragment through a flexible linker to enhance the stability and half-life of GDF15 [9]. In preclinical studies, AMG-171 demonstrated marked weight loss in obese mice, rats, and cynomolgus monkeys, with a greater reduction in fat mass compared to lean mass [9]. Furthermore, AMG-171 was found to improve metabolic parameters, including blood glucose, serum insulin, and triglyceride concentrations [9]. The therapeutic outcomes were attained in the absence of harmful consequences linked to GDF15 up-regulation in other vital organs, including the brain, heart, lungs, and liver [9]. However, AMG-171 has terminated its projects after phase 1 clinical trials due to lack of efficacy, resulting in a non-active status [9].

#### JNJ-9090/CIN109

CIN-109 is a long-acting analog of GDF15 for the treatment of obesity. In the completed phase 1 multiple ascending dose (MAD) study of CIN-109, marked weight loss was observed at all dose levels, with overall good tolerability in obese but otherwise healthy participants who completed the study. At the highest tested dose, the weight loss was primarily attributed to a reduction in fat mass, accompanied by an increase in lean body mass. No treatment-related serious adverse effects were noted. CIN-109 is a phase 2 ready candidate, and further clinical results are highly anticipated [188].

#### YH34160

YH34160, a GDF15 variant–Fc fusion construct, is optimized to strengthen receptor binding (GFRAL/RET), prolonging PK persistence and amplifying therapeutic potency [189]. Based on PK data from rodent and primate studies, YH34160 is anticipated to achieve an optimized PK profile in humans, allowing for once-weekly dosing [189]. Preclinical evaluations in DIO mouse models revealed that YH34160 induced pronounced and durable reductions in body weight [189]. Notably, when combined with GLP-1 receptor agonists or GLP-1/glucose-dependent insulinotropic polypeptide receptor agonists, YH34160 achieved more potent and marked weight loss compared to monotherapy mice.

#### Compound H

The long-acting GDF15 analog compound H, administered weekly in obese cynomolgus monkeys, achieved sustained reductions in food intake and peak body weight loss, markedly outperforming the GLP-1 analog dulaglutide [190]. Exposure-response modeling revealed that the weight loss effect of compound H was driven solely by reduced food intake rather than increased EE, with compound H ’s linear PK (~8-d half-life) supporting its clinical translation potential [190]. The study predicts that this long-acting GDF15 analog could achieve double-digit percentage weight loss in human obesity treatment.

#### The GLP-1/GDF15 dual agonist QL1005

QL1005 is an innovative GLP-1/GDF15 dual agonist, engineered through the fusion of GLP-1 and GDF15 analogs connected by a peptide linker and conjugated to a fatty acid for extended action [131]. GLP-1 receptor agonists are already widely used for their ability to reduce appetite, slow gastric emptying, and improve glycemic control, leading to marked weight loss in patients with obesity and type 2 diabetes [191]. Although this effect of GLP-1 agonists (liraglutide) or GDF15 is mainly attributed to reduced food intake, the combined treatment of these 2 proteins unexpectedly further reduced the body weight of mice. In vitro studies have shown that QL1005 possesses superior potency compared to the GLP-1 analog semaglutide [131]. In obese mice and cynomolgus monkey models, QL1005 induced dose-dependent reductions in body weight, food intake, insulin resistance, and metabolic parameters such as triglycerides and cholesterol with limited incidence of gastrointestinal side effects [131]. Notably, studies with variant compounds lacking either GLP-1 or GDF15 activity have highlighted the contribution of both arms of QL1005 to their overall pharmacological effects [131]. These studies suggest that the full therapeutic benefit of QL1005 is dependent on its dual agonistic activity.

Whether QL1005’s remarkable metabolic effects observed in animals will translate into impacts on humans warrants further investigation.

### GDF15 inhibitors

#### Ponsegromab

Despite the large number of cachectic patients, there is currently no effective treatment available for this condition. Ponsegromab, a humanized monoclonal antibody, is designed to neutralize GDF15 by binding to it with high affinity, thereby preventing its interaction with GFRAL [192]. In an open-label phase 1b study involving cancer cachexia patients with elevated GDF15 levels, ponsegromab was associated with improved weight, appetite, and physical activity [192]. These improvements were accompanied by a reduction in serum GDF15 levels [192]. The incidence of adverse events was low, suggesting good tolerability of the drug [192]. In a phase 2 trial involving 187 patients with cancer cachexia and elevated GDF15 levels, ponsegromab enabled patients to regain body weight within 12 weeks, with improvements in appetite, physical activity levels, and skeletal muscle mass [193]. Future research should continue to explore the combination of targeted therapies and immunotherapies to more effectively combat cancer cachexia and its related symptoms.

Ponsegromab’s ability to target GDF15 positions it as a versatile therapeutic candidate for a range of disorders. Clinical trials about heart failure (clinical trial no. NCT05492500) and cachexia (clinical trial no. NCT05546476) are ongoing. A recent study indicated that blocking GDF15 with ponsegromab mitigated cognitive deficits induced by sepsis, suggesting its potential as a therapeutic target for sepsis-associated encephalopathy [194].

#### Others

Several humanized neutralizing antibodies targeting GDF15, such as H53E5-8V2, LBL-049, and GFS202A, have demonstrated preclinical promise, yet their clinical profiles remain inadequately documented in peer-reviewed literature. H53E5-8V2 demonstrated robust binding affinity and superior efficacy to ponsegromab in inhibiting GDF15 signaling, along with favorable PK value in mice and efficacy in mitigating weight loss in preclinical models [195]. Similarly, LBL-049 exhibited high specificity for human and cynomolgus monkey GDF15 without cross-reactivity with other TGF-β family members, effectively blocking GDF15/GFRAL signaling in vitro and showing dose-dependent prevention of weight loss in cachexia models, outperforming ponsegromab at lower doses [196]. Additionally, GFS202A, a bispecific antibody targeting both GDF15 and interleukin-6 (IL-6), has shown preclinical efficacy in cancer-induced cachexia models in mice and cynomolgus monkeys, although detailed clinical data remain undisclosed, with available information limited to corporate announcements.

## GDF15: Friend or Foe?

GDF15 represents a fascinating yet complex hormone with context-dependent biological effects. This stress-responsive signaling molecule communicates bodily distress to the brain through its specific receptor GFRAL-RET, primarily influencing appetite regulation and potentially creating aversive responses. Its evolutionary significance likely stems from protective functions, particularly in toxin avoidance—a mechanism that persists in modern humans and may extend to pregnancy-related protective adaptations.

The hormone’s clinical relevance stems from its paradoxical roles: conditions of nutritional excess (e.g., obesity and diabetes mellitus), and up-regulated GDF15 expression may serve as a compensatory cytoprotective mechanism, maintaining homeostasis through negative energy regulation. In terminal disease states (e.g., advanced malignancies), elevated GDF15 levels may precipitate metabolic decompensation, as the disease progression exceeds the regulatory capacity of metabolic remodeling, with energy restriction precipitating cachexia syndrome and accelerating clinical deterioration. Given the bidirectional impact of the effects of GDF15 in different diseases, immunological intervention strategies require differentiation: Agonism of the GDF15–GFRAL pathway reduces caloric intake for obesity management, whereas antagonism of this pathway preserves normal energy intake for treating anorexia, cancer cachexia, and chemotherapy-induced adverse effects. Furthermore, the use of currently available GDF15 analogs necessitates meticulous dose control to balance efficacy with side effects such as vomiting.

## Concluding Remarks and Future Directions

As an endocrine hormone, the discovery of GDF15 and its receptor GFRAL has laid the foundation for target-based new drug research and development, opening up new ideas and directions for precision medicine. GDF15, a pleiotropic protein, plays a crucial role in various complex diseases and has emerged as a novel biomarker for disease diagnosis, progression, or prognosis, as well as an entirely new therapeutic target.

Although existing evidence has clearly established the role of GDF15 as an anorectic peptide, other physiological functions of the GFRAL-RET signaling pathway remain to be elucidated. Colchicine induces hepatocytes to express GDF15, which suppresses myeloid cell activation by positively regulating SHP-1. The absence of GFRAL expression in myeloid cells suggests that GDF15 may act through alternative peripheral mechanisms [32]. Furthermore, studies in hepatocellular carcinoma have identified CD48 as the first GDF15 receptor discovered within the immune system [197]. Protein expression of GFRAL and RET is detectable in peripheral blood platelets isolated from healthy volunteers, which provides evidence for the presence of GFRAL in the periphery [198]. The interplay between GDF15 and other appetite-regulating hormones warrants further investigation, as deeper insights could enhance drug safety profiles and reveal new immunotherapeutic targets. However, the physiological roles of the GDF15–GFRAL–RET axis and its implications in human diseases remain poorly understood, with many fundamental questions unresolved.

Regarding its precise anorectic and immune checkpoint functions, anti-obesity drugs have been developed by exploiting its activating effects, such as GDF15 analogs and GFRAL receptor agonists. Conversely, anorexic syndrome and anticancer drugs have been developed by leveraging its antagonistic effects, including GDF15 monoclonal antibodies and GFRAL antagonist monoclonal antibodies. However, the role of GDF15 in cardiovascular diseases, diabetes, metabolic liver diseases, and other disorders remains unclear and controversial, with conflicting research results, thus necessitating more evidence to support these findings. Furthermore, gender factors should be proactively incorporated into the design of future research to comprehensively evaluate their impact on study findings. In the future, personalized treatment plans based on the expression and presentation of GDF15 immune checkpoints, as well as in-depth research into *Gdf15* gene polymorphisms, may break through the bottlenecks in new drug development and achieve precision medicine for diseases.

The precise tissue and cellular sources driving elevated circulating GDF15 in cardiometabolic diseases remain incompletely resolved, reflecting context-dependent and species-specific regulation. While murine models highlight hepatic and adipose origins during metabolic stress, human data reveal paradoxes—notably the absence of obesity-associated *GDF15* mRNA up-regulation in adipose tissue despite elevated systemic levels [9,11]. Emerging evidence implicates multiple stress-responsive reservoirs. Critically, the relative contribution of these sources likely varies by disease state, tissue-specific stress burden, and interspecies differences in GDF15 biology. For example, in aged mice, *Gdf15* expression remained unchanged in liver, white/brown adipose tissue, kidneys, and heart, but exhibited discernible elevation specifically in skeletal muscle [199]. Integration of single-cell RNA sequencing, spatial transcriptomics, and secretomics will assist in revealing the tissue source of GDF15 under diverse cardiometabolic disease context.

In light of the biphasic effects of GDF15, a critical and objective analysis of its pharmacological actions is essential. While GDF15 exerts effects on pleiotropic effector organs, there may also be potential unanticipated adverse reactions. The development of GDF15-related drugs with activating or antagonistic effects remains challenging. During preclinical studies and clinical trials, while observing the clinical efficacy of the drugs, special attention should be paid to the adverse reactions they may induce.
